# Multiple Instance Learning for WSI: A comparative analysis of attention-based approaches

**DOI:** 10.1016/j.jpi.2024.100403

**Published:** 2024-10-20

**Authors:** Martim Afonso, Praphulla M.S. Bhawsar, Monjoy Saha, Jonas S. Almeida, Arlindo L. Oliveira

**Affiliations:** a Instituto Superior Técnico, Universidade de Lisboa, Av. Rovisco Pais, Lisbon 1049-001, Portugal; b Division of Cancer Epidemiology and Genetics, National Cancer Institute, National Institutes of Health, Bethesda 20850, MD, USA; c INESC-ID, R. Alves Redol 9, Lisbon 1000-029, Portugal

**Keywords:** Whole slide image, Multiple instance learning, Attention mechanism, Cancer, TP53

## Abstract

Whole slide images (WSI), obtained by high-resolution digital scanning of microscope slides at multiple scales, are the cornerstone of modern Digital Pathology. However, they represent a particular challenge to artificial intelligence (AI)-based/AI-mediated analysis because pathology labeling is typically done at slide-level, instead of tile-level. It is not just that medical diagnostics is recorded at the specimen level, the detection of oncogene mutation is also experimentally obtained, and recorded by initiatives like The Cancer Genome Atlas (TCGA), at the slide level. This configures a dual challenge: (a) accurately predicting the overall cancer phenotype and (b) finding out what cellular morphologies are associated with it at the tile level. To better understand and address these challenges, two existing weakly supervised Multiple Instance Learning (MIL) approaches were explored and compared: Attention MIL (AMIL) and Additive MIL (AdMIL). These architectures were analyzed on tumor detection (a task where these models obtained good results previously) and TP53 mutation detection (a much less explored task). For tumor detection, we built a dataset from Lung Squamous Cell Carcinoma (TCGA-LUSC) slides, with 349 positive and 349 negative slides. The patches were extracted from 5× magnification. For TP53 mutation detection, we explored a dataset built from Invasive Breast Carcinoma (TCGA-BRCA) slides, with 347 positive and 347 negative slides. In this case, we explored three different magnification levels: 5×, 10×, and 20×. Our results show that a modified additive implementation of MIL matched the performance of reference implementation (AUC 0.96), and was only slightly outperformed by AMIL (AUC 0.97) on the tumor detection task. TP53 mutation was most sensitive to features at the higher applications where cellular morphology is resolved. More interestingly from the perspective of the molecular pathologist, we highlight the possible ability of these MIL architectures to identify distinct sensitivities to morphological features (through the detection of regions of interest, ROIs) at different amplification levels. This ability for models to obtain tile-level ROIs is very appealing to pathologists as it provides the possibility for these algorithms to be integrated in a digital staining application for analysis, facilitating the navigation through these high-dimensional images and the diagnostic process.

## Introduction

Whole slide imaging is the automated process of digitally scanning whole microscope slides with high resolution. During this process, images from each field of view at different resolutions are taken and joined together to create a single digital image pyramid file, known as a whole slide image (WSI).^5^ This digital format, supported by a number of standard serializations, facilitates their distribution for diagnostic, education, and research purposes.^3^ In particular, WSIs play a critical role in cancer diagnosis.^6^

Deep learning has been successful in medical imaging applications such as diagnosis,^7^ sub-type classification,^6^^,^^14^ and prognosis.^25^ However, the application of deep learning on WSI faces three main challenges^9^: handling large image dimensionality at multiple scales; the lack of strongly annotated data; and more generally, the difficulties inherent to approaching classification with information retrieval.

Regarding the first factor, the large dimension of the images across multiple scales in the WSI image pyramid starts with resolutions at the base in the order of 100,000 × 100,000 pixels. The sheer size makes it difficult to feed the images directly as input to computer vision models. To overcome this issue, slides are usually divided into multiple fixed-size patches (also described as tiles) that are then used as input to the models.

The second factor is that the labeling is usually performed at slide level (for the whole WSI, not for individual tiles) or even at the patient level (one label per patient, which can have multiple slides). Expert labeling at the pixel level would be costly and exhausting to the pathologist. Without pixel-level annotations, fully supervised approaches cannot be directly employed and require instead weakly supervised^7^^,^^13^^,^^23^ or unsupervised^15^^,^^21^ approaches such as multiple instance learning (MIL),^2^ used in the work reported here.

Finally, it can be challenging to retrieve the relevant pathology information from relatively unstructured clinical reports. As a consequence, the interpretability of regions of interest (ROIs) identified by deep learning models is often poor to the point that attempting explanation at the morphology level can only be approached as an exploratory exercise. However, the ROIs approach, where recurring morphological patterns are observed, is a familiar procedure in Digital Pathology, where it is sometimes described as “virtual staining”.

In this work, we perform a comparison of two different attention-based MIL approaches (Attention MIL (AMIL)^7^ and Additive MIL (AdMIL)^8^) in two different tasks: tumor detection and TP53 mutation detection. We design a pipeline for preprocessing WSI from TCGA^22^ into patches and use it to build two distinct datasets for these tasks.

For tumor detection, we used slides from TCGA-LUSC (lung squamous cell carcinoma). Lung carcinoma was not explored for tumor detection with these architectures in their original works. Attention-MIL only explored breast cancer slides in their original paper and although AdMIL was tested with TCGA-NSCLC (non-small cell lung carcinoma), it was trained for sub-type classification. Furthermore, a different process was used for producing the weakly supervised datasets used. Therefore, we chose slides from TCGA-LUSC to perform a new and fair comparison between these models. This cancer type also contains a high amount of slides in TCGA, which makes it a good candidate for building a dataset for a deep learning task.

For our second task, we chose to approach *TP53* mutation detection. The *TP53* gene provides instructions for the production of a crucial protein with the same name, being responsible for suppressing tumors and exerting a central role in preventing the development of cancer. Mutations in the *TP53* gene can result in dysfunctional behavior and are linked to an elevated susceptibility to cancer. Therefore, algorithms capable of identifying possible mutations of this gene can be helpful for cancer diagnosis. For this task, we chose to evaluate the performance of the models at different magnifications (5×, 10×, and 20×). We do this in order to compare how different encoded information (from tissue-level to cell-level) influences the performance of weakly supervised deep learning models on this task. We chose slides from TCGA-BRCA (invasive breast carcinoma) because this cancer type had a fair amount of slides in TCGA and a relatively balanced distribution of positive/negative cases.

Besides the comparison of the two models in the tasks mentioned above, we changed one of the MIL architectures and performed further comparisons with the two initial approaches. Finally, we “stain” the raw images with heatmaps produced using the deep-learned activation scores resultant from the models in order to show the potential of attention-based MIL approaches for ROI detection and to provide a future evaluation and comparison of the models by pathologists.

## Materials and methods

### Multiple Instance Learning

Multiple Instance Learning (MIL)^2^ is a weakly supervised learning approach. The standard MIL definition uses a bag of instances as $X=\left\{x_{1},\ldots ,x_{K}\right\}$ that do not have dependency nor ordering among each other. For each bag *X*, there is a binary label *Y* ∈ {0,1}. Each instance within the bag has a label *y*_*i*_, where *y*_*i*_ ∈ {0,1}. Whereas the label for the entire bag is known during training, the labels for the individual instances remain unknown.

In the case of WSI analysis, we can consider each slide as a bag that contains several patches as instances. We have slide-level labels (bag label), but we do not have pixel/patch-level labels (instance labels).

To provide some degree of interpretability, as well as better results, a variation of the attention mechanism can be used as a MIL pooling operator. Because it acts as a weighted average of instances, the original definition can be adapted to be permutation-invariant, making it a valid MIL pooling operator. These attention scores can be used to build a heatmap that allows for the interpretation of which parts of an image are responsible for the final classification. AMIL^7^ was the first, to our knowledge, to apply an attention mechanism to the MIL problem.

Javed et al.^8^ mention that the AMIL approach still presents some problems regarding what is being interpreted in the image, because the attention scores might not be expressive enough for the explainability of the results. They noted that the non-linear relationship between the attention values and the final prediction makes the visual interpretation inexact and incomplete, stating that the attention scores obtained are not necessarily representative of tumors, but might still be needed for the prediction downstream, meaning they might be necessary but not sufficient for the final classification.

They propose an alternative additive architecture in order to address this issues with AMIL. In the AdMIL architecture, scores are generated for each patch after the attention mechanism, using a trainable layer. This scores represent the contribution of each patch (that can be either positive or negative) for the final classification. The scores are then added to produce a final bag score. AdMIL was explored for two tasks: cancer sub-type classification in a NSCLC dataset and renal cell carcinoma (RCC) dataset, and for metastasis detection in breast cancer using the Camelyon16 dataset.

Li et al.^12^ mention that the attention mechanism often focuses only on the most discriminative patches, resulting in a very sparse heatmap where only a few positive instances have high attention values. They use a technique known as Hide & Seek,^10^ where instances are randomly dropped during training (the pixel values of said instances are set to the mean RGB values of the dataset), forcing the network to discover new relevant instances instead of focusing only on the most discriminative ones.

An alternative attention mechanism, with a dual-stream MIL network, was also proposed.^11^ The first stream identifies the most critical instance with an instance classifier on their embeddings, whereas the second stream calculates an attention score based on the distance of each patch to the critical instance in the embedding space. It was trained specifically for WSI tumor detection. The authors propose two possible approaches: a single-scale approach that focuses on one-scale (20× magnification), and a multi-scale approach that concatenates the embeddings from patches at multiple scales (5×, 10×, and 20×).

MIL approaches that use self-attention mechanisms were also proposed. Li et al.^13^ propose an induced self-attention MIL architecture for medical image classification. Rymarczyk et al.^19^ also propose the integration of a self-attention mechanism in their work, where they explore the use of multiple kernels as a replacement for the dot product. Whereas this works achieved in general better results for the cohorts explored, these approaches are not as lightweight as AMIL or AdMIL. Due to the additional complexity and computational costs that come with self-attention mechanisms, these models might be difficult to integrate into lightweight WSI analysis applications. This is especially true for higher magnifications, because the number of patches increases exponentially.

Most works that use MIL focused, to our knowledge, on tumor and metastasis detection. Gene mutation detection was not as explored. Wang et al.^24^ trained ResNet models to predict the gBRCA mutation in breast cancer in different magnifications (5×, 10×, 20×, and 40×). The models were trained based on manually annotated tumor locations and the gBRCA mutation status. The cases for their dataset were collected from two medical centers in China. They reached the conclusion that the information captured at 5× magnification is less relevant for gBRCA prediction than higher magnifications. While achieving good results, this approach is only feasible when manual annotations are available.

Reisenbuchler et al.^17^ developed a local attention graph-based transformer for multi-genetic mutation prediction. By restraining self-attention to local regions of the WSI, the authors achieved state-of-the-art results for a colon cancer dataset (TCGA-CRC) and a stomach cancer dataset (TCGA-STAD). The model also allows for better interpretation, with attention scores calculated for the entire image. All images were downsampled to 20× magnification. The work from Guo et al.^4^ proposes a robust and lightweight AMIL algorithm for predicting genetic alterations. They use a K-means cluster algorithm to separate the patches into four groups according with their morphological features. Then, after choosing the cluster with the best predictive performance, they employ a lightweight attention mechanism for MIL. They compare their method with a self-attention approach, as well as four other weakly supervised approaches in fout cancer datasets from TCGA (UCEC, BRCA, GBM, and KIRC). We were not able to find which magnifications were used in their work.

### Model architectures

Due to the need for interpretability of the results, when choosing and building the models, we focused on MIL models that use an attention pooling mechanism. Because we also prioritized efficiency, we chose models with a relatively simple architecture. Specifically, we focused on the original AMIL, proposed by Ilse et al.^7^ and the AdMIL, proposed by Javed et al.^8^

Ilse et al. proposed a weighted average of the instances as a MIL pooling operator, where the weights were trained on a two-layered neural network. This operator corresponds to a version of the attention mechanism where all instances are independent.

In this approach, we have a 3-part model *g*, defined as:

(1) $$g\left(x\right)=\left(p\;\circ \;a\;\circ \;f\right)\left(x\right),$$

where *f* is the feature extractor for each instance *x*_*i*_ of the bag *x* with *n* instances, that returns the respective embedding vector *h*_*i*_, where $h_{i}\in h=\left[h_{1},\ldots ,h_{n}\right]$:

(2) $$h_{i}=f\left(x_{i}\right).$$

*a* is the attention module, that returns for each embedding vector *h*_*i*_ an attention score scalar. It is defined as:

(3) $$a_{i}\left(h_{i}\right)={\text{softmax}}_{i}\left(\psi_{a}\left(h\right)\right)h_{i}.$$

The term *p* is the predictor function or bag classifier:

(4) $$p\left(h\right)=\psi_{p}\left(\sum_{i=1}^{n}a_{i}\left(h_{i}\right)\right).$$

This term passes the sum of all embedding vectors multiplied by their respective attention score scalars through a final bag classifier ψ_*p*_. Both ψ_*a*_ and ψ_*p*_ are multi-layer perceptrons.

The attention mechanism is similar to Badhanau's attention,^1^ with the main difference that the instances are considered sequentially independent:

(5) $$\psi_{a}\left(h_{i}\right)=w^{T}\tanh\left(Vh_{i}^{T}\right)$$

with *w* as a weight vector and *V* as a weight matrix. Both are trainable parameters.

Javed et al.,^8^ as mentioned in Multiple Instance Learning proposed a new additive method for better visual interpretability and patch classification. To better specify the contributions from each patch, the final prediction function of the model described in eq. 4 undergoes the following change:

(6) $$p\left(h\right)=\left(\sum_{i=1}^{n}\psi_{p}\left(a_{i}\left(h_{i}\right)\right)\right),$$

where ψ_*p*_ produces a score for each patch. With this change, ψ_*p*_(*a*_*i*_(*x*)) becomes the contribution for patch *i* in a bag, turning it into a more accurate proxy of an instance classifier. The patch scores are then added to produce a final bag-level score, which in turn is converted to a final probability distribution using a *softmax*, to obtain the final classification. For interpretability and slide inpainting, the patch scores obtained can be passed through a *sigmoid* function to produce a bounded patch contribution score between 0 and 1, where 0–0.5 values represent inhibitory scores, and 0.5–1 values represent excitatory scores.

AMIL was originally trained with each WSI patch as bag (where each instance is a portion of that patch) for tumor detection, whereas AdMIL was trained for cancer sub-type classification and metastasis detection on WSI patches. The application of these models for tasks such as tumor detection and gene mutation detection can provide insights into the models' ability to generalize for different medical tasks. Furthermore, the differences in their employment of the MIL framework make it worthwhile to study and understand how these models focus on the different regions of slides.

**Fig. 1:**
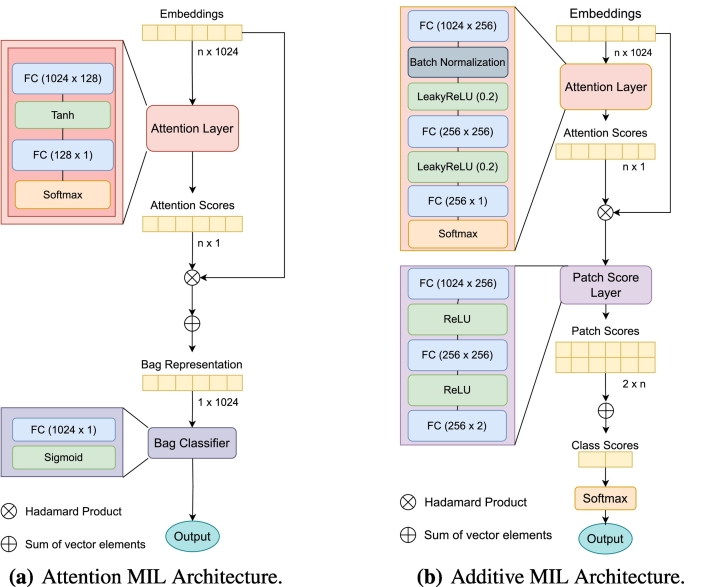
Models' architectures. The attention layers are composed of fully-connected (FC) layers, followed by activation functions. The AMIL (a) uses a tanh as its activation function, whereas the AdMIL (b) uses LeakyReLU. At the end of the attention layers, the results for each embedding are passed to a softmax to produce the final attention scores.

The models' original architectures used in this work are represented in Fig. 1. The original AMIL model is composed of a feature extractor module, followed by the attention module which produces attention scores for each instance, and the final bag classifier module which takes the sum of all instances multiplied by their respective scores and outputs the final bag classification. Similarly to the AMIL architecture, the AdMIL model is composed of a feature extractor module and an attention module. However, as mentioned before, instead of aggregating the patch embeddings through a weighted sum and then passing the result through a final bag classifier, AdMIL passes each patch embedding multiplied by its attention score through a patch classifier, the Patch Score Layer. This classifier produces a patch score for each class. These scores are not bounded by any values and can be either negative logits (inhibitory scores) or positive logits (excitatory scores). In the end, the scores are added together to produce a final bag score that is passed through a softmax, returning the final probability for each class.

By comparing the attention scores produced by the two models, we noticed that the use of the LeakyReLU activation function on the attention layer of the AdMIL architecture produced narrower attention scores.

The use of LeakyReLU as the attention layer's activation function can lead to a more skewed distribution of attention scores. This is because LeakyReLU allows larger positive values to pass through, which can later dominate when passed through a softmax function, leading to a few regions getting significantly higher attention scores. Therefore, the ROIs detected by the LeakyReLU-based attention mechanism tend to be more focused and localized. The model tends to identify more specific and concentrated areas as critical, potentially ignoring broader regions with lower attention scores.

This might not be ideal. Whereas pinpointing specific critical areas might be needed for some tasks, we should also consider to identify broader regions. On the other hand, the tanh function compresses the input into a relatively narrow range, which tends to produce attention scores that are more spread out. Therefore, this activation function is less likely to produce highly sparse attention scores because it does not suppress values close to zero. Instead, it spreads attention somewhat uniformly unless the input values are highly distinct. With this reasoning, we decided to experiment on an additional model that uses the architecture of AdMIL, but with the attention layer of AMIL.

In this study, we focused on the comparison of these two lightweight architectures, that used distinct strategies for producing patch scores. However, further comparisons and analysis could be made with other architectures referred in section 2.1. Furthermore, while we decided to compare the models as they were defined in their original works, we lacked the time to perform fine-tuning on the models' layers. These aspects are left as future work.

### Datasets

We chose two projects from TCGA^22^ to be analyzed in this work: TCGA-LUSC for the tumor detection and TCGA-BRCA for the TP53 mutation detection.

For the tumor detection task, we only used flash-frozen slides. Even though frozen specimens are less suitable for computational analysis when compared with formalin-fixed paraffin-embedded (FFPE) slides, we decided to build our dataset with these because of the lack of FFPE slides in the TCGA containing only healthy tissue. For this task, we focused on 5× magnification tiles. We chose this magnification level for its lower number of tiles per slide (and therefore, less storage consumption) and because at this level, patches show tissue-level information, which is more adequate for tumor detection tasks. This is supported by the fact that this is the magnification level at which pathologists analyze WSI for tumor detection, as confirmed by pathologist co-authors of this article.

For TP53 mutation detection, we used FFPE slides, because the unbalanced distribution was no longer a problem and these slides provided better results and training performance. Furthermore, the feature extractor we used for the tiles, KimiaNet,^18^ was trained with FFPE slides, so conforming to the same would lead to better results. For this task, we built three datasets at three different magnification levels: 5×, 10×, and 20×, in order to better understand at which magnification the models could best detect correlations between the mutation and the morphology of the tissue.

Due to the large size of FFPE slides and to save storage space and time, we performed a random sampling of tiles for these slides, depending on the magnification level. Moreover, whereas slide-level tumor presence labels were available for the first task, gene expression labels are only available at case-level (patient-level), presenting some challenges. We assumed that not only will the mutation be present in all diagnostic slides from a patient labeled as positive, but also that it would cover enough tissue to be captured in the tiles sampled in our dataset.

#### TCGA-BRCA

The TCGA-BRCA is composed of 1098 cases. It contains 1133 FFPE slides and 1978 flash-frozen slides across those cases. This dataset was used for the TP53 mutation task. We focused on the mutations of the *TP53* gene because it shows a greater number of mutated cases from those tested for simple somatic mutations (331 of 969 cases), allowing us to build a balanced dataset. For this task, we have 349 positive slides and 670 negative slides. We chose an equal number of positive and negative WSIs, and after filtering inadequate slides through the processing pipeline, we ended up with a total of 662 slides, 331 labeled positive, and 331 labeled negative.

#### TCGA-LUSC

The TCGA-LUSC is a dataset for lung squamous cell carcinoma. The size of this dataset is fairly small when compared with TCGA-BRCA, with 504 cases, containing 512 FFPE slides and 1100 flash-frozen slides across those cases. For the tumor detection task, we have 753 positive slides and 347 negative slides. Its class distribution for the tumor detection task is not too imbalanced for our purposes. We chose an equal number of positive and negative slides, ending up with a dataset composed of 694 slides. Its reduced number of slides, as well as the presence of more artifacts makes this type of cancer more challenging to work with. The TCGA-BRCA slides always have a tumor percentage of 90% at least, whereas in the case of TCGA-LUSC, the distribution of tumor percentage is more balanced. Therefore, we decided that for this task, a dataset built with slides from TCGA-LUSC is preferable for validating the ability of the model to generalize with different slides and tumor percentages.

### WSI preprocessing pipeline

WSIs are often too large to be directly processed by deep learning models. Slides can occupy over 6 GB of memory, so having a dataset of raw slides on disk is not feasible in our case. To store our dataset locally, we decided to focus on one magnification at a time. All our models were trained in separate magnification levels, which guaranteed that this would not be an issue. For some magnifications, the total of patches might exceed the storage space available. To overcome this issue, we developed a pipeline that fetches WSI tiles, processes, filters, efficiently encodes them, and saves the resulting embeddings and relevant metadata on HDF5 files. This pipeline is explained in the following sections.

#### WSI metadata extraction

We start by extracting information about each slide: id, labels type of slide, and microns per pixel (mpp) at which the slide was originally scanned. In the end, all the metadata extracted is saved to a CSV (comma-separated values) file for further processing.

#### Patch fetching and pre-processing

WSIs can have a lot of patches consisting exclusively of background or artifacts that make them unusable for training our models. Fetching all these unnecessary patches and checking them one by one becomes inefficient and time-consuming. To avoid this, we take advantage of WSI's multi-scale property to avoid fetching patches that will definitely contain only background.

The tile at the thumbnail level is initially fetched. Otsu's thresholding^16^ is applied to it, as well as a close morphological operation to filter noise. We then extract the resulting black pixel coordinates, obtaining a set of pixels *P*, the pixels that correspond to tissue.

In the acquisition of a WSI, there are often unintentional artifacts due to manual tissue preparation, staining, and scanning hardware, as well as pathologists' annotations. To mitigate the number of tiles containing artifacts as well as remove some background pixels that might have passed through the previous filter, the color of each pixel *p* ∈ *P* was compared with the average color of *P*, by calculating their Euclidean distance and comparing it with a pre-defined threshold. The coordinates of the pixels that fulfilled this condition were then stored and used to calculate the corresponding coordinates of the patches at the desired magnification, using the hierarchical properties of WSI and the metadata extracted from the step in 2.4.1.

Because these filters were applied at the thumbnail level, some of the tiles were still not suitable for the final dataset. After fetching each tile, we checked if its size in image coordinates was 512 × 512 px. If it was smaller, it was padded accordingly with the average background color. The percentage of tissue present in the tile was then calculated and compared with a threshold. If it does not contain enough tissue, the image is discarded.

**Fig. 2:**
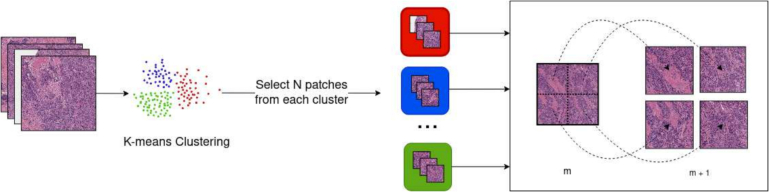
Sampling method for 10× and 20× magnifications. K-means clustering is applied to the set of tiles chosen from the previous magnification *m*. *N* tiles from each cluster are selected and the corresponding tiles at the magnification desired (*m* + 1) are then fetched.

For TP53 mutation detection, due to the size of the FFPE slides and the multiple magnification levels used, we applied random sampling to the tiles:

• At 5× magnification, we sampled 60% of the filtered tiles, when their number was greater than a certain limit; otherwise, we used all the tiles.
• In the case of 10× and 20× magnification, we applied clustering on the tiles from the previous magnification and sampled a chosen number n of tiles per cluster. We then proceeded to use the hierarchical properties of WSI to extract the corresponding tiles at the desired magnifications. For instance, we performed K-means clustering on the tiles at 5× magnification, sampled a maximum of 20 tiles from each cluster, and proceeded to extract, for each tile, the corresponding tiles at 10× magnification (Fig. 2).

#### Feature extraction

As mentioned previously, embeddings were created from the tiles. For this, we chose KimiaNet,^18^ a Densenet-121 pre-trained for WSI tumor subtype classification, that produces embedding vectors with a length of 1024. This model was trained exclusively on FFPE slides.

We also performed two data augmentations per tile, composed of random HED stain perturbation, Gaussian noise addition, rotations, and horizontal and vertical flips. Embeddings were generated for these augmented tiles as well. The embeddings are then saved to an HDF5 file, along with the corresponding relevant metadata, such as the coordinates of the patches and labels. This file can then be quickly read to memory during the training process of the models.

Due to the size of the dataset, immediately converting each 512 × 512 pixels patch to an embedding of length 1024 saves storage space (with a decrease in size close to a 1000-fold) and time for training the model and allows us to have the whole dataset locally, instead of having to fetch the data each time we needed to train or fine-tune the models. Furthermore, by having the slides represented in feature space immediately, as opposed to pixel space, we were able to fit all patches in a slide into GPU memory concurrently, which is especially useful for MIL approaches.

### Training methods

We split each dataset into a training set (80%) and a test set (20%). For each task and model, the training set was used to perform 5-fold cross-validation in order to prevent overfitting. The test set was then used for external validation, with the model extracted from the fold with the best results. For a final evaluation, we performed five runs, each one with a different seed for splitting the training set and test set. The results of these runs were used to calculate an average of the metrics and their standard deviation of the resulting areas under the curves (AUCs). For all the models, the loss function used was the binary cross-entropy loss.

## Results

### Classification and ROI detection results

In this section, we present the results obtained for our classification tasks, as well as some examples of the ROIs produced by the models.

Table 1, Table 2 display the average and standard deviation of the AUCs for the test set obtained from the five runs mentioned in section 2.5. We also present graphs displaying the ROC curves of one of these runs for each model, task and magnification.

**Table 1:** Models' performance for the tumor detection task on TCGA-LUSC. After hyperparameter fine-tuning, we performed five independent runs, each one with a different seed for splitting the training and test set and calculated the average test set AUC of those five runs for each model. The original architecture of AMIL obtained the best performance, followed by our version of AdMIL.

Model architecture	AUROC
Attention MIL (AMIL)	0.971±0.015
Additive MIL (AdMIL)	0.955±0.012
Our Additive MIL	0.963±0.020

**Table 2:** Models' performance for the TP53 mutation detection task on TCGA-BRCA for the three magnification levels explored (5×, 10×, and 20×). After hyperparameter fine-tuning, we performed five independent runs, each one with a different seed for splitting the training and test set and calculated the average test set AUC of those five runs for each model. The AMIL architecture always obtained the best performance. In general, the models obtained a better performance for higher magnification levels, with our version of AdMIL obtaining a better performance than the original at magnification level 20×.

Magnification level	Model architecture	AUROC
5×	Attention MIL (AMIL)	0.605±0.020
	Additive MIL (AdMIL)	0.575±0.038
	Our Additive MIL	0.512±0.034
10×	Attention MIL (AMIL)	0.711±0.033
	Additive MIL (AdMIL)	0.624±0.046
	Our Additive MIL	0.547±0.045
20×	Attention MIL (AMIL)	**0.704±0.040**
	Additive MIL (AdMIL)	0.624±0.033
	Our Additive MIL	0.642±0.029

Table 1 shows the AUC results for the tumor detection task at magnification 5×. Fig. 7 shows an example of the heatmaps produced for the tumor detection task in a slide at the same magnification level. Table 2 presents the TP53 mutation detection task's AUC results at magnification levels 5×, 10×, and 20×. Fig. 8, Fig. 9 present examples of heatmaps produced for the TP53 mutation detection task from patches of magnification 10× and 20×. Due to the poor classification results obtained at magnification 5×, we did not include heatmaps from this level, because we concluded that they would not be meaningful.

For the AMIL model, we only have the attention scores, corresponding to the patches that were considered the most relevant for the final prediction. Regarding the original AdMIL and our version, we have the patch attention scores produced by the attention layer, as well as the excitatory and inhibitory patch scores, that indicate positive and negative contributions for the final prediction, respectively. These final scores were passed through a sigmoid to scale the logits to values between 0 and 1, where values in the interval [0, 0.5] indicates a negative contribution and values in [0.5, 1] a positive contribution. In the case of attention heatmaps, we show a continuous colormap. For the inhibitory/excitatory scores, we only use two colors, one for the excitatory patches (Red) and other for the inhibitory patches (Blue).

## Discussion

### Tumor detection task

The models performed on par with the state-of-the-art for this task, in terms of their ability to correctly classify WSI, with AUC values above 0.9 (Table 1). The original article^7^ used two H&E image datasets: the breast cancer dataset and the colon cancer dataset. Comparing our results with the ones reported in this work, we obtained significantly better results than the first and similar results to the second. We should note, however, that the original datasets are not only from a different cancer type but also composed of patches as bags, instead of slides. Other works used the AMIL architecture and its own variations with datasets composed of tiles at higher magnifications.^11^^,^^20^ In general, we obtained a better AUC than previous reported work, which supports the fact that 5× magnification might be adequate for identifying tumors.

Considering that the dataset we used only had flash-frozen slides, that it presented some artifacts, and that it included varied percentages of tumors, we can assume that the models learned to differentiate unrelated factors from the tumors present in the images, although we cannot access the influence that possible artifacts might have had for the final results. Moreover, these results were obtained from tiles at 5× magnification, a level that displays tissue but not cells. This supports the hypothesis that, just like pathologists, models can learn to identify tumor slides at the tissue level. It also reveals that the task itself might not be challenging enough, and exploring higher magnifications will not meaningfully improve the slides' classification ability. It was due to these results and, as mentioned in section 2.3, the fact that this is the most common level used for tumor detection by pathologists that we did not explore further magnification levels for this task.

By analyzing the attention scores produced, we observed that both the AMIL Architecture (Fig. 7b and our version of the AdMIL model (Fig. 7f) produce sparser attention scores when compared with the original AdMIL (Fig. 7d) as we previously predicted. This behavior might be especially important if the tumor regions (or the desired ROIs) appear more scattered. However, for morphologies that are usually more focused on a specific patch, the attention layer from the original AdMIL could be preferable.

On the other hand, when analyzing the excitatory/inhibitory scores produced by the AdMIL framework, the scores produced by the original AdMIL model tend to be positive for most of the patches (Fig. 7c), whereas the ones produced by our version of AdMIL only highlight a small portion (Fig. 7e). This behavior was observed for most of the slides. Because the only difference in the models' architecture is their attention layers, we conclude that sparser attention scores tend to make the instance classifier consider only a small subset of patches as excitatory. Depending on the results acquired from a future evaluation of the heatmaps' relevance, this might support the hypothesis that attention scores do not necessarily highlight the desired ROIs and might not be enough for meaningful WSI painting.

It is also interesting to notice that the AMIL attention scores tend to focus more on the edges of tissue (Fig. 7b). This might mean that the tumor is present in the borders of the tissue, but it might also reveal some bias in the model for patches that include some percentage of background. Due to the magnification used, and the fractures that appear in flash-frozen slides, there was a significant amount of patches that contained background, when compared with higher magnifications and it is possible that this conditioned the model's learning ability.

However, further evaluation of the ROI detection needs to be done, with a focus on a qualitative evaluation of the heatmaps obtained. In order to do this, we would need the assistance of pathologists to confirm that the patch scores that these models obtain are indicators of tumor regions. Furthermore, whereas the AMIL model showed a slightly better AUC than the other models, this does not necessarily mean that its heatmaps are more relevant or helpful for pathologists. The patch scores obtained from the other two models, due to their inhibitory/excitatory nature, might provide greater insight into tumor presence in the slides. A manual evaluation of the heatmaps, as well as the production of a ground-truth for ROIs is, however, a laborious and time-consuming task for pathologists. Due to time constraints and the non-existence of ground-truth heatmaps, this evaluation is left as future work. Therefore, although we provide examples of heatmaps, in this article, we highlight the comparison of the models in terms of their quantitative results (AUCs obtained) as the main contribution.

### TP53 mutation detection task

For the magnification level 5×, the models' performance was poor (Table 2). The AMIL model showed the best performance, with an average AUC of 0.605. The hypothesis that was already supported by the results in previous work^24^ is still supported here: tiles at 5× magnification, a level at which only tissue is visible, cannot show discernible evidence of TP53 mutations for most cases. Therefore, regarding the model's ability to identify mutations for this gene, it is not possible to draw conclusions from this magnification level.

At level 10×, we obtained far better AUC results (Table 2). The AMIL model still shows the best performance in terms of its average AUC (0.711), but the AdMIL model also presents a reasonable score (0.624). Our version of AdMIL, however, did not improve considerably for most runs, with an average score of 0.547.

For this task, at this level, the number of patches highlighted in heatmaps is far scarcer. In the case of the AMIL model, unlike the previous task, the attention mechanism focused more on the inner patches (Fig. 8b). This could be due to the fact that it is looking for a different pattern in the morphology that might not be present in the borders, but it is also true that, at these magnification levels, the number of patches with background is scarcer, unlike the previous task.

The original AdMIL model focused on a very small number of patches (Fig. 8c). Even though it classifies slides as showing signs of TP53 mutation, at this magnification level, it does not seem to be able to point to relevant regions, only highlighting individual patches that are distant and isolated from each other. Just like in the tumor detection task, its attention mechanism only focused on a small number of patches (Fig. 8d).

Our version of AdMIL, on the other hand, is unable to identify any excitatory patches from the majority of WSI (Fig. 8e). This might have to do with the fact that its attention mechanism is not able to make a notable distinction between patches, attributing very similar attention scores to all of them (Fig. 8f). This also explains the low average AUC obtained by this model at this magnification level.

At level 20×, the results did not change by much for AMIL and AdMIL, but improved a lot for our modified version of AdMIL, with an AUC score of 0.642. For this level, the heatmaps produced also did not show much change from the ones at magnification 10×. However, it is interesting to notice that the attention scores produced by our version of AdMIL, unlike the previous level, focused around a specific region on the WSI (Fig. 9f). However, this model continues to produce only inhibitory scores.

The generated heatmaps do not seem to highlight a significant number of patches. This might be because signs of TP53 mutation might appear more isolated in these slides. However, further evaluation by specialists would have to be done to reach a definite conclusion.

Therefore, as we did with the tumor detection task, we highlight the analysis of our quantitative results. The AUC scores obtained by our models for levels 10× and 20× are comparable with the ones obtained by previous work,^4^^,^^17^ although we have used simpler architectures. However, this comparison should be taken carefully, because the models were trained on different datasets (with different methods for tile pre-processing) and often for a different type of gene mutation. In general, the quantitative results for this task are not as good as those from the tumor detection task. In fact the most noteworthy quantitative results are the ones from AMIL.

We speculate that the models' ability to learn patterns related to the TP53 mutation might have been affected by the dataset used. Although we only used FFPE slides, which are typically better for WSI computational analysis (and KimiaNet was trained with this type of slides), the fact that, due to time constraints, our WSI bags were built using random sampling might have reduced the quality of their representation of the slide. Furthermore, we assumed that all slides belonging to a positive patient are positive as well. All these factors might have introduced too much noise in the dataset.

Beside the possibility of too much noise, we come to the conclusion that detecting patterns of TP53 mutation on digital slides with MIL and attention mechanisms is a much more challenging task, and the results are not as good as when detecting the presence of tumors.

## Conclusions

**Fig. 3:**
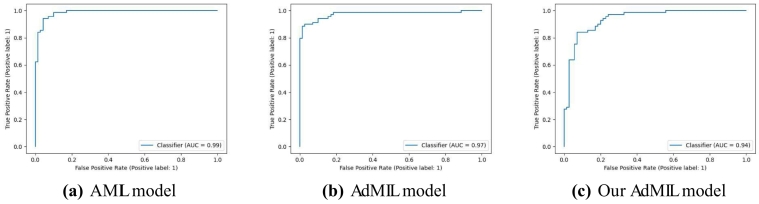
ROC curves for one of the runs of the tumor detection task (5× magnification) on TCGA-LUSC.

**Fig. 4:**
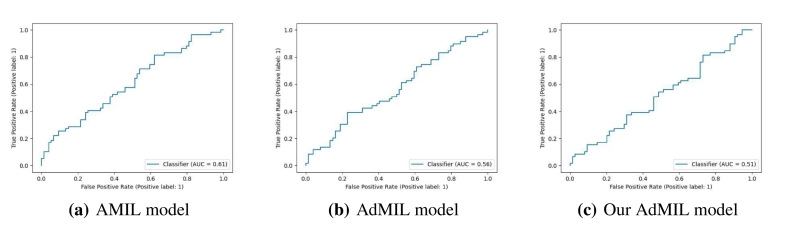
ROC curves for one of the runs of the TP53 mutation detection task (5× magnification) on TCGA-BRCA.

**Fig. 5:**
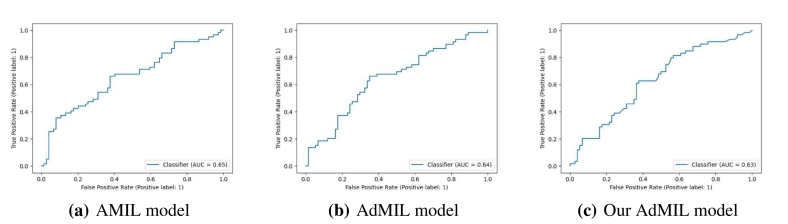
ROC curves for one of the runs of the TP53 mutation detection task (10× magnification) on TCGA-BRCA.

**Fig. 6:**
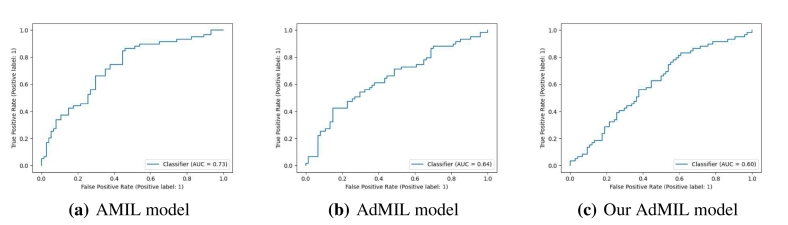
ROC curves for one of the runs of the TP53 mutation detection task (20× magnification) on TCGA-BRCA.

In this work, we study the performance of MIL frameworks with attention mechanisms for WSI classification and virtual staining. We compared two distinct approaches that identify different morphology proxies for patch (tile) classifiers. These two frameworks were used in a weakly supervised application to tumor detection in LUSC and TP53 mutation detection in breast carcinoma. We found that it was far easier to identify ROIs recognizing tumor vs non-tumor even at low resolution (AUC > 0.95), than it was to classify TP53 mutated vs non-mutated (AUC < 0.71). In general, the original AMIL architecture had the best quantitative performance. The observation that higher resolutions (10× and 20×) worked better to classify and identify ROIs for mutation was by itself not a surprise, but the opportunities to hypothesize novel morphological interpretations emerged as the main result from the work reported here. Similarly, when we explored new modifications of the established MIL method by altering its original attention layer, the most interesting result was not improved accuracy but the difference in the attention the model placed on different morphological features (i.e., “slide painting”). Specifically, the results described in Fig. 3, Fig. 4, Fig. 5, Fig. 6 illustrate the opportunities for interactive exploration of recurring morphologies for their role in cancer etiology.

**Fig. 7:**
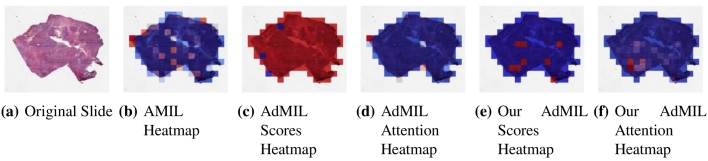
Heatmaps produced for slide 05194610-c32d-44db-be55-c38652d547b8 (TCGA-LUSC) for the tumor detection task at 5× magnification. (a) Slide at thumbnail level; (b) AMIL attention scores; (c) AdMIL's final patch scores; (d) AdMIL's attention scores; (e) our version of AdMIL's final patch scores; (f) our version of AdMIL's attention scores.

**Fig. 8:**
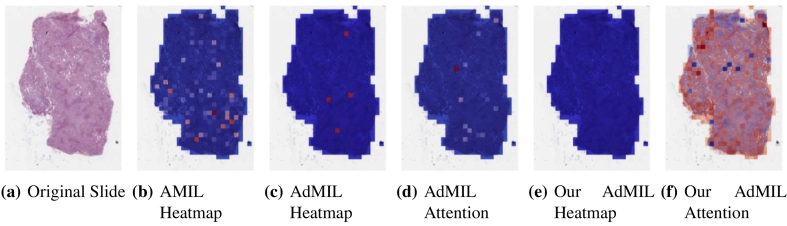
Heatmaps produced for slide d42eb4ce-d300-46be-8b46-059a98c2ddb8 (TCGA-BRCA) for the TP53 mutation detection task at 10× magnification. (a) Slide at thumbnail level; (b) AMIL attention scores; (c) AdMIL's final patch scores; (d) AdMIL's attention scores; (e) our version of AdMIL's final patch scores; (f) our version of AdMIL's attention scores.

**Fig. 9:**
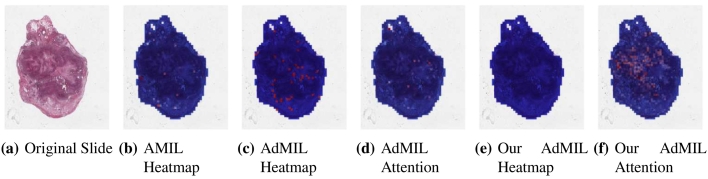
Heatmaps produced by the models for slide cf1ee3be-f161-4614-b2dd-030a9adfc5fc (TCGA-BRCA) for the TP53 mutation detection task at 20× magnification. (a) Slide at thumbnail level; (b) AMIL attention scores; (c) AdMIL's final patch scores; (d) AdMIL's attention scores; (e) our version of AdMIL's final patch scores; (f) our version of AdMIL's attention scores.

As future work, we highlight the need for an evaluation done by experts on the heatmaps generated. Moreover, whereas we focused on the analysis and comparison of two MIL architectures that use distinctive patch score mechanisms, further comparisons can be done with the architectures developed in other works, mentioned in section 2.1. AMIL and AdMIL were also evaluated as they were defined in their original works, but further fine-tuning of their architectures can also be made.

## Declaration of competing interest

The authors declare that they have no known competing financial interests or personal relationships that could have appeared to influence the work reported in this article.
